# EHPnet: Consumer Action Guide to Toxic Chemicals in Toys

**Published:** 2008-02

**Authors:** Erin E. Dooley

On 5 December 2007, the nonprofit Ecology Center launched the Consumer Action Guide to Toxic Chemicals in Toys at **http://www.healthytoys.org/** to provide parents and other caregivers with information on toys containing potentially hazardous chemicals such as lead, arsenic, and mercury. The site currently contains basic screening information on more than 1,200 toys that were tested using X-ray fluorescence to determine amount of various chemicals on a toy’s surface. Each toy receives an overall rating as well as separate ratings for 5 chemicals—lead, chromium, chlorine/PVC, arsenic, and mercury. The ratings are not intended to measure health risk or actual user exposure.

The Product Action Guide lists toys by manufacturer and type and by best and worst rated; visitors can also search by specific toy name. Each toy’s page lists its ratings as well as an overview of which parts of the toy were tested, and the amounts of the chemicals of concern detected. Also noted is the presence of other potentially toxic chemicals such as tin, antimony, chromium, and bromine if levels above 100 ppm were found (these chemicals are not, however, used in calculating the overall rating for items).

The Buy Toys Safely page under the Take Action subhead offers advice for buying safer toys. The site also has a list of frequently asked questions that includes topics ranging from lead paint in homes to how to find out about product recalls. In the Chemicals of Concern section, visitors can learn about the chemicals tested, alternatives to these chemicals, and other resources for information on healthy products, toy safety, and ecolabeling programs in the United States and abroad. Visitors can also use the Test My Toy! feature to nominate additional toys for testing. In the site’s first 2 weeks, more than 4,500 additional toys were nominated, of which 22 were tested.

